# Metabolic Action of Metformin

**DOI:** 10.3390/ph15070810

**Published:** 2022-06-30

**Authors:** Izabela Szymczak-Pajor, Sylwia Wenclewska, Agnieszka Śliwińska

**Affiliations:** 1 Department of Nucleic Acid Biochemistry, Medical University of Lodz, 251 Pomorska Str., 92-213 Lodz, Poland; agnieszka.sliwinska@umed.lodz.pl; 2 Department of Internal Medicine, Diabetology and Clinical Pharmacology, Medical University of Lodz, 251 Pomorska Str., 92-213 Lodz, Poland; sylwia.wenclewska@umed.lodz.pl

**Keywords:** metformin, hepatic gluconeogenesis, glucose metabolism, lipid metabolism

## Abstract

Metformin, a cheap and safe biguanide derivative, due to its ability to influence metabolism, is widely used as a first-line drug for type 2 diabetes (T2DM) treatment. Therefore, the aim of this review was to present the updated biochemical and molecular effects exerted by the drug. It has been well explored that metformin suppresses hepatic glucose production in both AMPK-independent and AMPK-dependent manners. Substantial scientific evidence also revealed that its action is related to decreased secretion of lipids from intestinal epithelial cells, as well as strengthened oxidation of fatty acids in adipose tissue and muscles. It was recognized that metformin’s supra-therapeutic doses suppress mitochondrial respiration in intestinal epithelial cells, whereas its therapeutic doses elevate cellular respiration in the liver. The drug is also suggested to improve systemic insulin sensitivity as a result of alteration in gut microbiota composition, maintenance of intestinal barrier integrity, and alleviation of low-grade inflammation.

## 1. Introduction

Currently, the incidence of type 2 diabetes (T2DM) is becoming an epidemic, and the treatment of complications caused by chronic hyperglycemia is extremely economically burdensome. Chronic hyperglycemia exerts a direct toxic effect on different cell types, including pancreatic β-cells and vascular endothelial cells. Specifically, in the insulin resistance state preceding symptomatic T2DM, prolonged hyperglycemia contributes to oxidative stress that is highly dangerous to β-cells. Moreover, as a result of increased secretion of insulin, β-cells become exhausted and die. Thus, secretion of insulin is disturbed. In turn, vascular endothelial cells are particularly sensitive to hyperglycemia since they transport glucose in an insulin-independent manner, and intracellular glucose concentration is proportional to its blood concentration. Thus, endothelial cells are directly exposed to the toxic effect of high glucose and related oxidative stress, which leads to micro- and macro-vasculature dysfunction, initiating the development of diabetes complications in multiple organs that significantly affect the length and quality of life. It was demonstrated that metformin action is limited not only to the decrease in hyperglycemia, but also to a delay in the development of diabetic complications [1,2,3]. The mechanisms of metformin’s action are complex and associated with multiple targets in the body [4,5]. Therefore, the aim of the review was to provide updated knowledge concerning the molecular and biochemical actions of metformin involved in metabolism regulation.

## 2. The Fate of Metformin in the Human Body

After oral ingestion, metformin is absorbed by proximal small-intestine enterocytes. It has been proposed that passive diffusion is responsible for 50% of metformin uptake in the intestine. This type of transport is conducted by paracellular and transcellular pathways. However, there is no agreement whether paracellular, transcellular, or both pathways are engaged in metformin transport [6]. In addition to passive diffusion, the rest of the drug is transported by facilitated diffusion employing numerous transporters. The primary transporter involved in metformin’s absorption is plasma membrane monoamine transporter (PMAT) present on polarized enterocyte apical membranes. The affinity of PMAT to metformin determined by Michaelis constant (K_m_) is equal to 1.32 mM [7]. Another transporter found on the apical membrane of enterocytes participating in metformin absorption is organic cation transporter 3 (OCT3) that possess a K_m_ of 1.10 mM. Due to the fact that OCT3 possesses a lower K_m_ for metformin, its affinity to the drug is higher as compared to PMAT. This was confirmed by Chen et al., who reported that the deletion of OCT3 evoked decreased metformin bioavailability and circulating level [8]. The transport of metformin into the portal vein also occurs through OCT1 found on the basolateral membrane of enterocytes [9]. Several other transporters such as serotonin transporter (SERT), thiamine transporter 2 (THTR2), and carnitine/organic cation transporter 1 (OCTN1) participating in the intestinal absorption of metformin have been also identified. However, their precise role in metformin absorption is not fully known.

It was observed that the drug is undetectable in the plasma for 24 h after oral administration of single dose, and its half-life of elimination is equal to 7.2 h [10]. Plasma metformin levels in the portal vein range from 40 to 70 µM in animals after administration of a therapeutic dose. As a result of the transport of metformin with the blood, the drug is delivered directly to the liver, where its uptake is mediated by OCT1 and OCT3, achieving 3–5 times higher concentrations than in portal vein [11]. OCT1 and OCT3 are key transporters that take up the drug, since their knockout evokes significantly lower hepatic metformin accumulation and reduced suppression of glucose production [8,12,13]. As a result of hepatic uptake of metformin, its plasma concentration drops to 10–40 µM in both humans and animals [14]. The excretion of metformin from hepatocytes to circulation occurs through multidrug and toxin extrusion 1 (MATE1), and MATE inhibition causes the accumulation of the drug in the liver [15].

**Figure 1:**
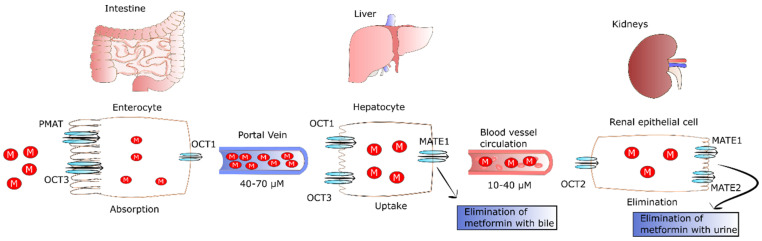
The fate of metformin in the human body. After oral ingestion, 50% of metformin is absorbed by passive diffusion, and the rest of the drug is transported by facilitated diffusion via PMAT and OCT1 transporters in intestinal enterocytes. Then, the drug leaves enterocytes via OCT1 and is transported to the liver via the portal vein where it reaches concentrations of 40–70 µM. Metformin enters the liver via OCT1 and OCT3 where it inhibits gluconeogenesis. The drug is not metabolized by the liver, but MATE1 expressed in hepatocytes participates in elimination of unchanged drug with the bile or in its transport with the blood to kidney. Then, metformin enters renal epithelial cells via OCT2. Next, the drug is secreted by renal MATE1 and MATE2 in unchanged form and eliminated with urine. Abbreviations: M, metformin; PMAT, plasma membrane monoamine transporter; OCT1, organic cation transporter 1; OCT2, organic cation transporter 2; OCT3, organic cation transporter 3; MATE 1, multidrug and toxin extrusion protein 1; MATE2, multidrug and toxin extrusion protein 2.

Metformin is not metabolized by the liver; however, MATE1 expressed in hepatocytes is involved in elimination of unchanged drug with the bile or in transport of metformin with the blood to kidney [10]. Renal excretion of the drug in unchanged form by tubular secretion into urine is the major pathway of metformin clearance [16,17]. The transport of metformin to the kidney is mediated by MATE1, MATE2, and OCT2. The latter is a key transporter for uptake of the drug by renal epithelial cells (K_m_ of 0.99 mM) [18]. In turn, MATE2 (K_m_ of 1.05 mM) and MATE1 (K_m_ of 0.23 mM) participate in metformin secretion from the tubule cells into the urine. Furthermore, MATE1 is also involved in the secretion of metformin into the bile [17,19,20,21]. The fate of metformin in the human body is presented in Figure 1.

Comparing the K_m_ of MATE1 and MATE2 of renal cells, one can observe that MATE1 has higher affinity to metformin than MATE2. Gan et al. revealed that MATE1 deletion evoked systemic elevation of metformin and lactic acidosis, the major side-effect of the drug. The pathophysiology of metformin-induced lactic acidosis is likely because of suppression of gluconeogenesis via blocking of pyruvate carboxylase (PC) [22]. The lack of PC activity results in the plasma accumulation of pyruvate that is next changed into lactic acid via lactate dehydrogenase (LDH), triggering its increased plasma concentration. LDH is an enzyme catalyzing the conversion of pyruvate into lactate and the reverse reaction that require NAD^+^ reduction and NADH oxidation, respectively. Hepatic transformation of lactate into pyruvate and muscle-specific conversion of pyruvate into lactate are closely related processes known as the Cori cycle or lactic acidic cycle that comprises gluconeogenesis and glycolysis. In the Cori cycle, anaerobic glycolysis-derived lactate is transported from the muscle to the liver where it is transformed into glucose. In turn, glucose returns to the muscles where it is metabolized to lactic acid [23,24,25]. Thus, the Cori cycle may be involved in metformin-induced lactic acidosis because the drug inhibits PC activity, leading to plasma accumulation of pyruvate. In turn, pyruvate participates in the Cori cycle and is transformed into lactic acid. At therapeutic concentrations of metformin, lactate is changed back to glucose in the Cori cycle. Conversely, metformin accumulation as a result of improper elimination or excessive drug intake leads to reduced hepatic lactate uptake and lactic acidosis. However, lactic acidosis and hyperlactatemia do not develop in all patients with isolated overdose of metformin [26].

## 3. Dose-Dependent and Tissue-Specific Effect of Metformin on Cellular Respiration

Cellular respiration involves a number of processes of combustion of organic compounds in which, in addition to carbon dioxide, reduced nucleotides such as FADH_2_ and NADH are formed. Some of these processes take place in the cytoplasm (i.e., glycolysis), while some take place in the mitochondria (i.e., β-oxidation and citric acid cycle). The last steps of cellular respiration, namely, respiratory chain and oxidative phosphorylation producing water molecules and ATP, occur in the mitochondria [25].

The rate of cellular respiration is strictly associated with mitochondrial biogenesis. This process is characterized by mitochondria growing in both size and number within the cell. Thus, it is crucial for sufficient combustion of organic compounds and ATP generation. Peroxisome proliferator-activated receptor γ coactivator-1α (PGC-1α) is a co-transcriptional regulation factor. PGC-1α activates mitochondrial biogenesis via inducing numerous transcription factors i.e., nuclear respiratory factors 1 and 2. In turn, these factors induce mitochondrial transcription factor A that governs both replication and transcription of mitochondrial DNA. PGC-1α is regulated via some factors, e.g., AMPK. AMPK, also called an energy sensor, controls respiratory chain activity and mitochondrial fission [27,28]. The maintenance of mitochondrial homeostasis during environmental and metabolic stress involves mitochondrial fusion and fission. Mitochondrial fission is responsible for the formation of new populations of mitochondria, elimination of damaged mitochondria, and induction of apoptosis. In turn, mitochondrial fusion reduces stress via the connection of some components of damaged mitochondria [29]. 

The effect of metformin on the cellular respiration is dose-dependent and tissue-specific. It was demonstrated that a supra-therapeutic doses of metformin caused a decrease in mitochondrial respiration only in intestinal epithelial cells, where its concentration was markedly higher in comparison to the circulation [16,30]. In order to maintain energetic homeostasis, intestinal epithelial cells accelerate glycolysis, leading to overproduction of lactate. The presented mechanism is suggested to be responsible for metformin-induced intestinal lactate production [31,32].

Hollinger observed that a supra-pharmacological dose of metformin (~20 mM) decreased oxygen uptake by mitochondria by 30% [33]. The molecular mechanism responsible for reduced mitochondrial respiration was proposed only in the early 2000s, showing that metformin induced suppression of complex I of the mitochondrial respiration chain in vitro [34,35]. This is in the line with previous observations concerning phenformin and other biguanides conducted over 50 years ago, reporting the inhibition of mitochondrial complex I activity [36,37,38]. In addition, it was also associated with diminished ATP generation [34,35]. However, it was also revealed that metformin (IC_50_ of 19–66 mM) presented weak suppression of mitochondrial respiratory chain complex I [39,40]. Owen et al. suggested that the inhibitory effect of the drug toward complex I may be connected with the storage of positively charged metformin molecules in the mitochondrial matrix driven by mitochondrial membrane potential [34]; however, this hypothesis was ultimately not confirmed [30]. It was shown that a supra-pharmacological dose of metformin elevated the mitochondrial membrane potential, accelerating the generation of ATP from ADP. However, when the ADP level was diminished, mitochondrial membrane potential could not be exploited to generate ATP. Wang et al. also reported that a supra-pharmacological dose of metformin pronouncedly decreased cellular ADP, resulting in decreased generation of ATP. Moreover, exogenous ADP is able to restore the mitochondrial respiration inhibited by high concentrations of metformin [41]. 

Some human studies have presented that therapeutic concentrations of metformin induced activity of mitochondrial respiratory chain in cells and tissues other than epithelial cells of intestines [42,43]. The results of studies involving mice also confirmed that a therapeutic dosage of the drug induced activity of mitochondrial respiratory chain complex I in the liver, and not only diminished hyperglycemia, but also increased the number of mitochondria in HFD mice [41,44]. T2DM patients are characterized by decreased numbers of mitochondria, resulting in diminished cellular respiration efficacy in the liver, and mitochondrial dysfunction is suggested as one of factors associated with T2DM development [45]. It was demonstrated that 75 μM metformin activated AMPK-induced phosphorylation of dynamin-related protein 1 (DRP1) and mitochondrial fission factor (MFF) resulting in elevated hepatic fission of mitochondria [27,41]. Hepatocytes derived from liver-specific Drp1^−/−^ mice presented markedly reduced mitochondrial respiration and elevated accumulation of fat [41]. Moreover, metformin treatment of Drp1^−/−^ mice evoked the elimination of damaged mitochondria in hepatocytes via mitophagy, which resulted in the maintenance of the normal mitochondrial population [41].

Karise et al. observed that metformin elevated the thermogenesis and biogenesis of mitochondria in the BAT of mice fed with high-fructose diet. These metabolically stressed mice presented metformin-induced strengthening of thermogenic markers (PCG-1α and UCP-1) via FGF-21 and adrenergic stimuli in BAT. Additionally, metformin elevated levels of mitochondrial biogenesis markers (TEAM and NRF-1). The drug also changed fatty uptake markers (CD36, LPL, and aP2) and lipolysis markers (HSL, ATGL, and perilipin) leading to improvement of fatty-acid uptake and lipolysis [46]. Geerling et al. showed that metformin increased VLDL-TG-related fatty-acid uptake by BAT of mice fed a Western diet. Moreover, although changes in both protein and mRNA UCP1 levels have not been reported, this effect coexisted with a markedly diminished content of lipids in BAT and reduced mass of animals [47]. These results [46,47] suggest that metformin elevated the biogenesis of mitochondria since the drug upregulated PCG-1α and increased mitochondrial respiratory chain capacity in BAT. Moreover, metformin mediated the increase in phosphorylation of AMPKα at position T172 in BAT, induced AMPK, and increased the activity of respiratory chain and mitochondrial fission [27,47], thus leading to elevated fatty-acid utilization. 

### 3.1. Metformin Inhibits Hepatic Glucose Production (Gluconeogenesis)

It is widely known that metformin lowers hyperglycemia by suppressing gluconeogenesis in the liver [17]. Wang et al. observed that a pharmacological concentration of metformin reduced cAMP-stimulated production of glucose by 58% in cultured primary hepatocytes [41]. This effect is a result of the metformin-driven suppression of glucagon. Glucagon, a hormone secreted by α-cells of pancreas, stimulates gluconeogenesis. In hepatocytes, glucagon binding with its receptor stimulates adenylate cyclase responsible for cAMP production. In turn, cAMP activates PKA engaged in glucose production. Miller et al. suggested that metformin inhibited adenylate cyclase as a result of decreasing ATP level and elevating AMP level. Thus, metformin impaired the glucagon signaling pathway in hepatocytes via inhibition of cAMP formation essential for PKA activation, thereby preventing gluconeogenesis [48,49]. In turn, Takashima et al. observed that gluconeogenesis decreased by over 60% in response to metformin using a euglycemic clamp test [50]. A metformin-mediated decrease in endogenous glucose production by 50% was also reported in HFD-fed rats [51]. Several other studies revealed that metformin inhibited hepatic gluconeogenesis by more than 33% in humans [52,53]. 

Inzucchi et al. observed ~20% reduced hepatic glucose production in response to metformin treatment among patients with improper control of T2DM [54]. Results of a placebo-controlled study confirmed an approximately ~15% decrease in hepatic glucose production after metformin treatment in poorly controlled T2DM subjects [55]. However, contradictory effects were observed by Gormsen et al. who presented a paradoxical elevated production of glucose in the liver in both recent-onset T2DM patients and nondiabetic subjects [56]. It was also proposed that the above-observed effect was related to a pronounced compensatory elevated level of glucagon in the plasma of nondiabetic patients [57].

### 3.2. The Molecular Background of Gluconeogenesis Inhibition Exerted by Metformin

#### 3.2.1. AMPK-Dependent

Mitochondrial respiratory chain complex I is involved in coupling the movement of electrons directed from reduced nicotinamide adenine dinucleotide (NADH) to ubiquinone with transmembrane pumping of protons generating the proton motive force required for the synthesis of ATP. In turn, this mitochondrial proton gradient and synthesized ATP are the energetic cost of gluconeogenesis. Metformin diminishes the electron transport chain activity, thus decreasing [ATP]:[ADP] and [ATP:AMP] ratios [35,48]. Thus, the lowered energy charge in hepatocytes activates AMPK and, in consequence, leads to suppression of gluconeogenesis [48,52,58]. Although this mechanism is universally recognized, it is contested due to the fact that it was identified in studies employing supra-pharmacological doses of metformin [59,60]. Additionally, some results of in vivo studies also questioned the ability of metformin to alter the hepatic energy charge without activating AMPK [61,62]. 

In turn, Miller et al. revealed that the elevated hepatic level of AMP induced by metformin treatment elicited an allosteric reduction in adenylyl cyclase activity, which resulted in decreased production of cAMP and antagonized the action of glucagon in the liver [48]. However, the results of other studies again did not confirm that metformin, by lowering cAMP level, was able to antagonize glucagon in clinically relevant doses [59,61,63]. Additionally, the results of a clinical trial involving prediabetes patients did not show an inhibitory effect of metformin on glucagon-dependent hepatic glucose production [57].

The speed of gluconeogenesis depends on fructose 1,6-bisphosphatase (FBP-1,6) [48]. In line with the allosteric-dependent mechanism, it has been documented that the AMP-independent regulation of the expression of a mutant fructose 1,6-bisphosphatase abrogated the metformin glucose-lowering effect in vivo [64]. 

Metformin-evoked inhibition of mitochondrial respiratory chain complex I, resulting in the elevation of AMP and AMPK activation. In the basal state, AMPK is bound to ATP. As a metabolic sensor, AMPK, in response to metabolic stress such as intense exercise or prolonged hunger, replaces ATP with AMP or ADP, leading to its allosteric activation [65,66]. AMPK activation involves phosphorylation at Thr172 mediated by Ca^2+^/calmodulin-dependent protein kinase β (CAMKKβ) or hepatic B1 kinase (LKB1) [65]. 

The proposed AMPK-dependent mechanism is related to the decreased expression of genes involved in gluconeogenesis, which decreases hepatic glucose production. This leads to the hepatic reduction in lipogenesis and induction of oxidation in mitochondria, consequently diminishing diacylglycerol content and enhancing insulin sensitivity in the liver [52,58,67,68]. 

Zhou et al. observed that activity of ACC was lowered, oxidation of fatty acids was activated, and expression of enzymes involved in lipogenesis was inhibited in metformin-treated rats [52]. Both ACC1 and ACC2 are molecules constituting key AMPK-activated targets. These molecules are engaged in lipid metabolism regulation because they catalyze the reaction that leads to malonyl-CoA production. In turn, malonyl-CoA is known as a de novo lipogenesis precursor that governs fat oxidation in mitochondria [69,70,71]. The suppression and phosphorylation of ACC2 and ACC1 via AMPK reduce lipogenesis and elevate fat oxidation in the liver [67], triggering a decrease in hepatic accumulation of lipid and an improvement in insulin sensitivity. The study on mice with ACC double knock-in, characterized by insensitivity to suppression of AMPK, presented that AMPK-dependent suppression of ACC was required for the metformin therapeutic actions in HFD-fed mice [68]. 

It is also known that AMPK suppresses gluconeogenesis via inhibition of some transcription factors, including CREB-regulated transcription coactivator 2 (CRTC2) and hepatocyte nuclear factor 4 (HNF4). These transcription factors stimulate the expression of some gluconeogenic enzymes i.e., G6PC and PCK1 [72,73]. Moreover, AMPK is able to reduce gluconeogenesis via induction and phosphorylation of the nuclear exclusion of class IIa histone deacetylases. These enzymes deacetylate and activate FOXO in the nucleus, promoting the expression of gluconeogenic enzymes during fasting [74].

Cao et al. documented that AMPKα1/2 subunit depletion abolished metformin-dependent suppression of glucose production in primary hepatocytes [63]. Further studies reported that liver-derived AMPKα1/2^−/−^ mice fed with an HFD diet were characterized by elevated levels of fasting blood glucose in response to metformin treatment for 12 weeks. Elevated production of glucose and increased mRNA levels of Pck1 and G6pc were observed in AMPKα1/2^−/−^ mouse-derived primary hepatocytes. Additionally, AMPKα1 or α2 plays a crucial role in metformin-dependent suppression of hepatic glucose production because AMPKα1 constitutes a key AMPK catalytic subunit [41]. Meng et al. showed that metformin action leads to hepatic AMPKαβγ heterotrimeric complex formation both in mice fed with HFD and in vitro. The metformin-induced AMPKαβγ complex triggers elevated phosphorylation at the Thr172 site of the α subunit via increased LKB1-dependent phosphorylation and suppression via protein phosphatase-mediated dephosphorylation [75]. Metformin was also observed to be able to promote the relocation of both LKB1 and AMPK to lysosomes, triggering to activation of AMPK [76]. Metformin was not able to stimulate AMPK and did not decrease hyperglycemia in hepatic LKB1^−/−^ mice fed with HFD [58]. 

Liver-derived LKB1^−/−^ mice were characterized by hyperglycemia, AMPK inactivation, and increased expression of genes involved in gluconeogenesis. Moreover, LKB^−/−^ mice were not sensitive to the metformin hypoglycemic effect, proposing that the LKB1–AMPK–CRTC2 pathway may be involved in metformin’s mechanism of action [58]. Opposite to these observations, the results of other study showed that LKB1^−/−^ hepatocytes presented response to therapy with metformin. Additionally, liver-derived AMPK deletion was not sufficient to inhibit action of metformin [61]. The inconclusive findings from the presented studies may be the result of different doses of metformin used, variation in metformin administration (i.e., intragastric or intraperitoneal), and type of diet (i.e., HFD or regular chow).

It was also found that metformin suppresses mitochondrial glycerol-3-phosphate dehydrogenase (mG3PDH) activity, leading to an elevated level of cytoplasmic NADH, as well as increased cytosolic redox state, contributing to metformin’s antihyperglycemic effect [59]. mG3PDH is negatively regulated via AMPK [17]. The presented mechanism is crucial in patients characterized by prominent lactate levels in the serum. Elevated NADH levels suppress the transformation of lactate to pyruvate, triggering reduced glucose production from lactate [77].

To sum up, the results of the presented studies clearly show that reduced gluconeogenesis evoked by metformin engages stimulation of LKB1–AMPK signaling [78].

#### 3.2.2. AMPK-Independent

Another mechanism of metformin-dependent action is the elevated cytosolic redox state as a result of inhibition of hepatic GPD2 activity [79,80,81]. GPD2 suppression decreases gluconeogenesis, in which redox-derived substrates including lactate and glycerol are involved. The presented selectivity of substrates for metformin-dependent suppression of hepatic gluconeogenesis was shown in the results of in vivo and in vitro studies [60,82]. No alterations in gluconeogenesis involving lactate after intervention with metformin was observed in rat liver [83]. On the contrary, pronounced suppression of GPD2 in response to metformin was observed [77]. The results of several studies presented that phenformin and metformin exert a suppressor effect on activity of GPD2 [59,84,85,86]. The observed effect also confirmed the results of in vivo and in vitro studies presenting elevated concentrations of glycerol and G3P in the liver in response to metformin [59,87].

Despite the fact that GPD2 expression is observed throughout the human body, its expression levels are tissue-dependent [88,89,90]. The presented findings were questioned by the results of studies suggesting that GPD2 mediates hepatic metformin action, partially because of the high pancreatic GPD2 expression level. Metformin was not found to suppress the secretion of insulin or inhibit GPD2 in the pancreas. However, these results did not consider the distribution of metformin within tissues. Metformin is primarily accumulated in the tissues (i.e., kidney, liver, and small intestine) as a result of the specific profile of MATE1, OCT3, and OCT1 transporter expression, needed for the uptake of metformin [91,92,93,94]. Moreover, metformin treatment was presented to change redox balance not only in the liver but also in the kidneys [59,95], consistent with the inhibition of GPD2 within metformin-accumulated tissues [96].

The suppression of the malate–aspartate shuttle was also proposed as a result of metformin action. This shuttle is a biochemical system responsible for translocating glycolysis-produced electrons across the semipermeable mitochondrial inner membrane for oxidative phosphorylation in eukaryotes. However, the proposed mechanism was questioned due to the lack of metformin influence on enzymes such as aspartate aminotransferase or malate dehydrogenase [59,77]. The malate–aspartate shuttle is responsible for counterbalance in the case of long-term alterations in redox balance; however, the influence of GPD2 suppression on the participation of glycerol in gluconeogenesis cannot be denied. Glycerol turnover is elevated in T2DM patients as a result of WAT-derived inflammation and insulin resistance, which trigger higher glycerol participation in gluconeogenesis [97,98,99]. Thus, it may be suggested that metformin is able to change redox balance even in cases where the glycerophosphate shuttle cannot surpass malate–aspartate shuttle rates [100,101,102].

The results of the studies carried out on GPD2^−/−^ mice, as well as in subjects characterized by deficiency and/or mutations of GPD2 have suggested new insight into the metabolic results of GPD2 suppression [59,103,104,105]. It was observed that GPD2^−/−^ mice are resistant to hyperglycemia induced by diet, independently of the insulin secretion stimulated by glucose [105]. Disturbances of glycerophosphate shuttle activity also suppress hepatic glucose production involving glycerol, triggering the perturbation of metabolism of lipids and amino acids in mice. From the clinical point of view, the importance of the presented alterations can be observed via the relationship between hepatic steatosis and decreased expression of GPD2 in subjects without and with NAFLD occurrence, suggesting decreased hepatic glucose production involving glycerol [104].

Although metformin reduces hepatic gluconeogenesis involving redox-dependent substrates via suppression of activity of GPD2, it is not fully understood whether the drug’s effect is indirect or direct [59,60]. The direct influence of metformin on GPD2 was reported in studies on mitochondrial lysates, intact mitochondria, and an isolated enzyme test with immunoprecipitated GPD2 [59,84,86]. 

Several hypotheses suggest an indirect GPD2 suppression by metformin. Metformin presents numerous effects and may change the efficiency of some electron transport chain complexes. These complexes indirectly trigger GPD2 suppression. Biguanides are able to bind metal ions, including iron and copper, by acting as a Schiff base. The presented effect is in line with reports showing direct binding of metformin with cytochrome c or heme [106,107,108]. Further studies are required to assess whether biguanides including metformin bind to iron and copper enclosed in the electron transport chain complex, which leads to GPD2 suppression. Interactions between metformin and metals may provide an explanation for the numerous effects exerted by metformin on the functions of the mitochondrion, as well as on metabolism.

### 3.3. How Does Metformin Act in Muscles, Adipose Tissue and Intestines?

#### 3.3.1. The Effect of Metformin Action in Intestine

Intestinal gluconeogenesis constitutes 5–7% of the systemic production of glucose [109], and enterocytes are the first cells exposed to metformin action [110]. It is well documented that supra-therapeutic doses of metformin inhibit the mitochondrial respiratory chain in hepatocytes [34,35,41], and concentrations of the drug are clearly higher in the intestine, thereby reducing the ATP level in enterocytes. Accordingly, to maintain a relevant cellular level of ATP, enterocytes enhance anaerobic glycolysis by producing lactic acid from glucose [31]. Metformin not only increases uptake of glucose from the intestinal lumen or circulation via relocation of GLUT-2 [111], but also elevates the usage of glucose in the intestine, generating lactate [31]. Lactate is utilized for glucose production in the liver, forming the intestinal liver cycle [32]. As a result of metformin’s suppression of the mitochondrial respiratory chain and AMP deaminase, the level of AMP in enterocytes increases. This in turn evokes the inhibition of adenylate cyclase and fructose-1,6-bisphosphatase 1 (FBP-1), consequently lowering the rate of gluconeogenesis in the intestine [48,64].

Glucagon-like-peptide 1 (GLP-1) is secreted by gut cells. This hormone governs meal-associated glycemic control via the elevated secretion of insulin and decreased secretion of glucagon. Metformin-mediated activation of AMPK increases GLP1 secretion by L cells and, thus, its circulation level in T2DM patients [112,113,114]. It was also observed that low concentrations of metformin increased the expression of GLP1 in L cells via sodium-dependent glucose cotransporter 1 (SGLT1) without activation of AMPK [115]. However, the results of studies aimed at determining the influence of metformin on secretion of GLP-1 production showed inconclusive results. On the one hand, direct influence on expression of GLP-1 was documented. On the other hand, an indirect influence driven by the activity of dipeptidyl peptidase-4 (DPP4) was also proposed. No significant effect on GLP-1 was reported [116,117]. Gontier et al. also revealed that the intestinal action of metformin comprises delayed emptying of stomach, increased secretion of GLP-1, and changed metabolism of glucose in enterocytes [118]. 

In addition to benefits related to intestinal glucose uptake, treatment with metformin is associated with gut microbiome composition and hormone (i.e., growth differentiation factor 15 (GDF15)) secretion alterations [31,110,119,120,121,122]. Elevated serum levels of GDF15 were found in T2DM patients in response to metformin treatment [123]. A potential mechanism responsible for the metformin-mediated induction of the integrated stress response pathway involves secretion of GDF15, which in turn contributes to improvement of glycemic regulation, as well as reduced appetite, via the hindbrain-situated receptor glial cell-derived neurotrophic factor family receptor alpha-like (GFRAL) [122,124]. In vivo, it was also observed that metformin induces both secretion and expression of GDF15 from hepatocytes. Coll et al. found that metformin markedly upregulated intestinal GDF15 expression without exerting an effect on hepatic GDF15 expression in metformin-treated mice [122]. Thus, further studies are needed to assess whether the liver is also engaged in metformin-mediated production of GDF15.

It is believed that undesired gastrointestinal side-effects such as nausea, diarrhea, constipation, stomach pain, decreased appetite, and weight loss reported by patients treated with metformin are the result of the intestinal glucose-lowering action of the drug [122,124,125]. 

#### 3.3.2. Muscle and Adipose Tissue

Both myocytes and adipocytes express OCT3; thus, these cells are also able to absorb metformin. Prolonged treatment of mice with metformin enhanced insulin-induced uptake of glucose in muscle [33,48,58,61,126], whereas inactivation of AMPK suppressed metformin-mediated glucose uptake [126]. It is also well documented that metformin increases the disposal of glucose and the contents of glycogen in skeletal muscles [127]. Moreover in visceral adipose tissue, the drug also elevates glucose uptake [46]. Generally, metformin was proven to increase whole-body glucose uptake in T2DM patients with obesity [24]. In poorly controlled T2DM patients, 3 month treatment with 1000 mg of metformin twice a day increased peripheral glucose uptake (~13%), decreased hepatic gluconeogenesis (~20%), and reduced fasting plasma glucose level (~58 mg/dL) [28].

The glucose uptake in skeletal muscle cells and adipocytes is induced by insulin and involves glucose transporters (GLUT). The molecular mechanism via which metformin increases peripheral glucose uptake in muscle cells is associated with the inhibition of SH2 containing inositol 5’ phosphatase (SHIP2) [128]. The enzyme is responsible for dephosphorylation of phosphatidylinositol 3,4,5-trisphosphate (PtdIns(3,4,5)P3) to phosphatidylinositol 4,5-bisphosphate (PtdIns(3,4)P2); thus, it suppresses insulin signaling. Metformin was demonstrated to block activity of SHIP2, thereby decreasing GLUT4 endocytosis and increasing glucose uptake in skeletal muscle cells [128]. Moreover, metformin-mediated AMPK stimulation inhibited PTEN, as well as strengthened insulin signaling, in 3T3 pre-adipocyte cells [129]. Lee et al. observed that AMPK knockdown reduced metformin-mediated formation of the Cbl/CAP multicomplex in 3T3-L1 preadipocyte cells. The Cbl/CAP–CrkII–C3G–TC10 pathway plays an important role in GLUT-4 translocation from the intracellular space to the cell surface. Thus, metformin stimulates the translocation of GLUT-4 via AMPK-dependent regulation of CAP and Cbl signaling in 3T3-L1 preadipocytes cells, improving glucose uptake in these cells [130]. Grisouard et al. demonstrated that AMPKα1 activation without attenuation of cell respiration is required for metformin-induced elevation of GLUT-4 protein level and uptake of glucose in human adipocytes [131]. In turn, Fischer et al. observed that metformin induced glucose uptake in preadipocyte-derived adipocytes. Moreover, the presented effect was not related to the source of preadipocyte fat deposits (visceral or subcutaneous) or obesity state of patients. It was proposed that metformin-stimulated glucose uptake is not dependent on insulin signaling in adipocytes [132].

Virtanen et al. observed decreased adipose tissue mass in response to metformin in T2DM patients [133]. Interestingly, metformin elevated the uptake of VLDL-TG–related fatty acids in the brown adipose tissue (BAT) of Western diet equivalent-fed mice. Metformin also markedly decreased the content of lipids and the mass of BAT without affecting the protein and mRNA levels of uncouple protein 1 (UCP1) [47].

## 4. Metformin Regulates Lipid Metabolism

### 4.1. Metformin Decreases the Secretion of Lipids from Intestinal Epithelial Cells

T2DM patients present abnormal metabolism of lipids, leading to significantly increased coexistence of fatty liver and cardiovascular diseases. On the other hand, metformin improves lipid metabolism, thereby reducing the risk of fatty liver and cardiovascular complications. This effect is partially connected to the property of metformin to decrease the concentration of chylomicrons in T2DM patients [134]. Metformin, via activation of AMPK and GLP1, diminishes the synthesis of apoA-IV and apoB-48. These are crucial mediators of chylomicron synthesis and secretion, and they are elevated in T2DM patients. The decreased level of apoA-IV and apoB-48 and reduced synthesis of triglycerides caused by metformin lead to a lowering of chylomicron formation, as well as secretion by enterocytes [41,112,113,114]. In addition, metformin was found to diminish cholesterol level in the circulation via a reduction in the reabsorption of bile acids in the intestine and an increase in chylomicron clearance [135].

### 4.2. Metformin as an Enhancer of Oxidation of Fatty Acids in Adipose Tissue and Muscles

**Figure 2:**
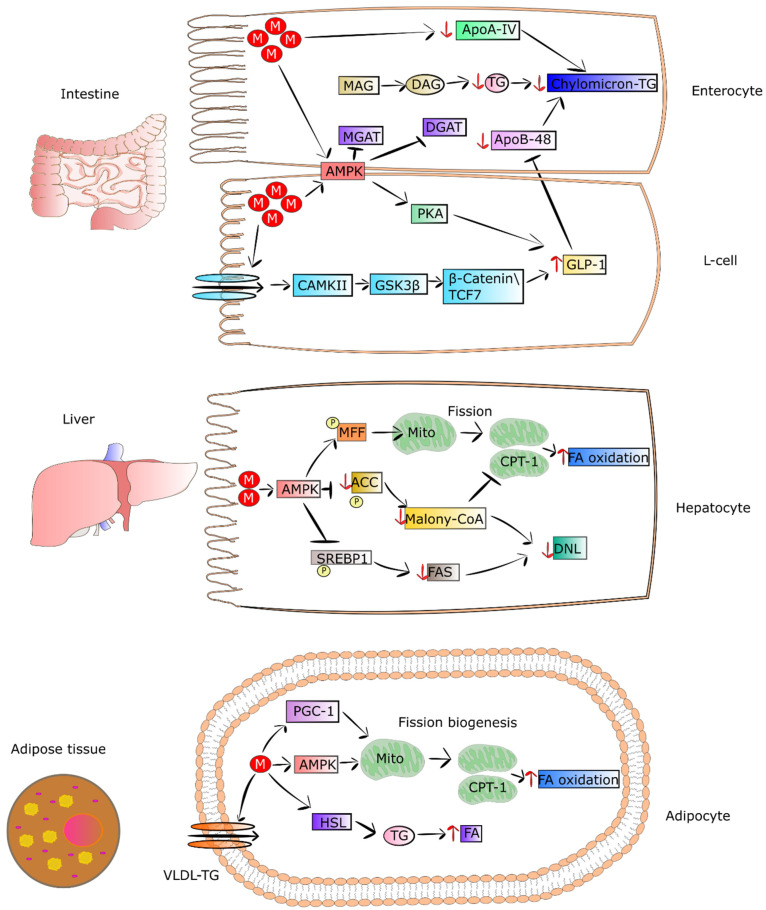
Metformin regulates the metabolism of lipids in enterocytes, L-cells, hepatocytes, and adipocytes. In the intestine, the drug initiates the expression of GLP-1 and its secretion via AMPK-independent and -dependent pathways in L cells. In enterocytes, metformin suppresses chylomicron storage and production via decreasing the levels of Apo-IV and Apo-48, as well as the synthesis of TG. In turn, in hepatocytes, metformin-dependent activated AMPK phosphorylates ACC and SREBP-1, leading to suppression of DNL and restoring CPT-1 activity. The drug also promotes the process of mitochondrial fission, leading to an increase in mitochondrial number and elevation of FA oxidation. The action of metformin in adipocytes contributes to increased uptake of FA and HSL activity, involving lipolysis. The drug also strengthens mitochondrial biogenesis via inducing PGC-1 signaling. Additionally, metformin activates AMPK, which also promotes mitochondrial fission. Lastly, the drug enhances oxidation of FA in adipocytes. Abbreviations: M, metformin; MAG, myelin-associated glycoprotein precursor; DAG, diacylglycerol; TG, triglycerides; ApoA-IV, apolipoprotein A-IV; ApoB-48, apolipoprotein B-48; MGAT, monoacylglycerol acyltransferase; DGAT, diglyceride acyltransferase; AMPK, 5’ adenosine monophosphate-activated protein kinase; PKA, protein kinase A; GLP-1, glucagon-like peptide-1; CAMPii, Ca^2+^/calmodulin-dependent protein kinase II; GSK-3β, glycogen synthase kinase-3 β; TCF7, transcription factor 7; MFF, mitochondrial fission factor; ACC, acetyl-CoA carboxylase; CPT1, carnitine palmitoyltransferase I; FA, fatty acid; FAS; DNL, de novo lipogenesis; SREBP1, sterol regulatory element-binding protein 1; malony-CoA, malonyl-coenzyme A; VLDL-TG, high-plasma very-low-density lipoprotein triglyceride; PGC1, peroxisome proliferation-activated receptor-gamma coactivator-1; HSL, hormone-sensitive lipase.

It was shown that metformin treatment of mice fed with HFD and T2DM patients exhibited loss of adipose tissue as a result of elevated uptake and utilization of fatty acids [46,133]. This led to a reduction in VLDL-TG level and lipid droplet content in BAT. Metformin pronouncedly elevates fatty-acid utilization and oxidative phosphorylation in the mitochondria via increasing the proteins involved in the mitochondrial respiratory chain. The drug was also reported to activate hormone-sensitive lipase (HSL) expression and phosphorylation of acetyl-coenzyme A carboxylase (ACC), AMPK, and HSL in differentiated adipocytes, thereby increasing lipolysis. It was also observed that metformin elevated both utilization and uptake of fatty acids in adipose tissue, which in turn may have been related to the decreasing in VLDL-TG and mass of adipose tissue in mice fed with HFD and patients [46,133]. Metformin-stimulated fibroblast growth factor 21 (FGF21) seems to be involved in the reduced mass of adipose tissue and elevation of fatty-acid oxidation in white adipocytes derived from obese mice. FGF21 is an important metabolic regulator participating in the control of lipolysis in WAT [136]. Wang et al. observed that metformin can suppress accumulation of fat via promoting fatty-acid oxidation in the skeletal muscle of ob/ob mice. Some genes engaged in fatty-acid oxidation and synthesis of acyl-CoA were downregulated including Ascl3, Ppard, Mlycd, and Acsbg1 [137]. The mechanisms by which metformin regulates metabolism of lipids in intestinal epithelial cells, muscles and adipose tissue are presented in Figure 2.

## 5. Other Metformin-Mediated Mechanisms Leading to Improvement of Systemic Insulin Sensitivity

### 5.1. Metformin Changes the Composition of Gut Microbiome and Maintains Intestinal Barrier Integrity

It is well recognized that patients suffering from T2DM and obesity show intestinal dysbiosis in comparison to healthy subjects [138,139]. In addition, HFD was demonstrated to increase the serum level of lipopolysaccharides (LPS), which in turn induced systemic inflammation, endoplasmic reticulum (ER) stress, and insulin resistance [140]. The results of studies performed on diabetic mice fed an HFD and treated with metformin revealed that the drug altered intestinal microbiota composition and improved insulin resistance [119,141]. Moreover, the transfer of metformin-changed intestinal microbiota to germ-free mice improved glucose tolerance. This was accompanied by a decrease in serum PLS level, alleviation of inflammation, and restoration of insulin sensitivity. In turn, in T2DM patients treated with metformin, an alteration in the composition and function of gut microbiome was detected [120]. It was observed that the proportions of genus *Akkermansia* and phyla Bacteroidetes, Bacteroides, and Verrucomicrobia were pronouncedly elevated in response to metformin treatment [142]. The presented metformin-induced alterations were related to a decrease in serum levels of LPS, alleviated inflammation, and improvement in insulin sensitivity.

Intestinal bile-acid sequestration was suggested as a kind of T2DM therapy. Metformin decreases bile-acid resorption. Thus, through the increase in the level of intestinal bile acids, metformin changes the intestinal microbiota composition [143]. Butyrate, propionate, and lactate, known as short-chain fatty acids (SCFAs) generated from glycolysis and the metabolism of amino acids and other organic compounds, are produced by intestinal microbiota. They constitute fuel for intestinal epithelial cells, thereby improving barrier integrity via AMPK induction [144]. Metformin treatment leads to a growth in intestine SCFA levels and AMPK activation, consequently strengthening the intestinal barrier integrity and triggering a decrease in LPS outflow. Eventually, his leads to a decrease in inflammation and insulin resistance in T2DM [119,120].

### 5.2. Metformin Decreases Systemic Low-Grade Inflammation 

**Figure 3:**
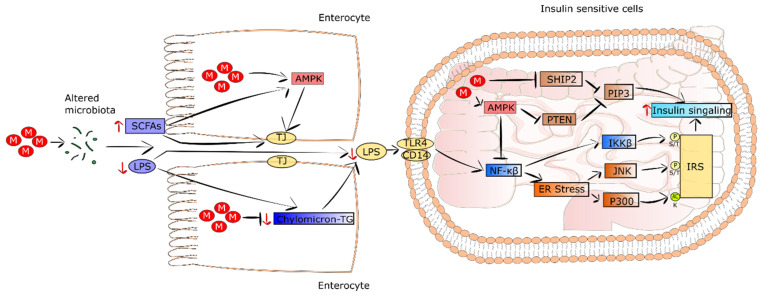
The improvement of systemic insulin sensitivity is a result of metformin-evoked microbiota alteration. In the intestine, the drug alters the microbiota composition, leading to reduced production of LPS and increased secretion of SCFAs. In turn, SCFAs and metformin-driven AMPK activation contribute to an improvement of the intestinal barrier, reducing the production of chylomicron, which in turn triggers a decreased level of LPS in circulation. In insulin-sensitive cells, the decreased level of LPS and low-grade inflammation lead to strengthening insulin signaling via decreased acetylation of IRS and phosphorylation of threonine and serine, as well as elevated PIP_3_ levels. Abbreviations: M, metformin; SCFAs, short-chain fatty acids; LPS, lipopolysaccharides; TG, triglyceride; TJ, tight junction; AMPK, 5’ adenosine monophosphate-activated protein kinase; TLR4, Toll-like receptor 4; CD14, cluster of differentiation 14; NF-κB, nuclear factor kappa-light-chain-enhancer of activated B cells; IKKB, inhibitor of nuclear factor kappa-B kinase subunit beta; SHIP2, SH2 domain-containing inositol 5-phosphatase 2; PIP3, phosphatidylinositol(3,4,5)triphosphate; PTEN, phosphatase and tensin homolog; ER, endoplasmic reticulum; JNK, c-Jun N-terminal kinase; IRS, insulin receptor.

The effect of LPS on insulin signaling was examined. It was revealed that LPS evoked an increase in acetyltransferase p300 protein level and its improper appearance in cytoplasm of hepatocytes [145]. This led to the acetylation of insulin receptor substrate ½ (IRS1/2), resulting in the inhibition of IRS1/2 binding to the insulin receptor, thus, a disturbance of insulin signaling. Metformin was found to augment IRS tyrosine phosphorylation in hepatocytes [139]. Moreover, LPS was reported to stimulate the NF-κB pathway and ER stress via JNK and IKKβ, causing serine/threonine phosphorylation of IRS and resulting in further impairment of insulin signaling. Contrarily, metformin via AMPK activation attenuated the ER stress and LPS-dependent induction of NF-κB, thereby decreasing insulin resistance of the liver [146]. Since the NF-κB pathway is engaged in proinflammatory cytokine expression, metformin treatment reduces the expression of TNF and CRP in T2DM patients Additionally, in the intestine, a supra-pharmacological concentration of metformin inhibited the differentiation of monocytes into macrophages via AMPK induction, triggering a decrease in proinflammatory cytokine secretion, as well as activating macrophage polarization into functional M2 phenotypes characterized by anti-inflammatory properties [147]. Thus, metformin improves insulin resistance not only by decreasing fat accumulation and inflammation in the liver, but also by reducing proinflammatory cytokines in circulation. The improvement of systemic insulin sensitivity as a result of metformin-evoked microbiota alteration is presented in Figure 3.

## 6. Conclusions and Future Perspectives

Metformin is a glucose-lowering drug which has been used for over 60 years. Despite such a long history of use and numerous studies conducted, the interest in metformin is still growing. This drug reduces hepatic glucose production in both AMPK-independent and AMPK-dependent manners. It has also been documented that metformin’s action is related to the diminished secretion of lipids from intestinal epithelial cells, as well as the increased oxidation of fatty acids in muscles and adipose tissue. Interestingly, therapeutic doses of metformin increase hepatic cellular respiration, whereas supra-therapeutic concentrations of the drug inhibit mitochondrial respiration in the intestine. Recent studies also identified a pronounced intestinal action of metformin, involving suppression of oxidative phosphorylation, increased glycolysis, and lactate overproduction. Other interesting aspects of how metformin works in the gut include its influence on the functions of endocrine cells and the composition of the gut microflora. These actions consequently reduce bodyweight and insulin resistance. Due to the multidirectional action of metformin, work is still underway to discover new properties of the drug, especially those connected to metabolism improvement, considering the current epidemic of metabolic diseases. 

## Data Availability

Not applicable.
